# Is tramadol associated to bleeding peptic ulcer? A nationwide case-control study in hospitalized Swedish patients

**DOI:** 10.1371/journal.pone.0215356

**Published:** 2019-04-17

**Authors:** Hans Järnbert-Pettersson, Marine L. Andersson, Katarina Bilén, Olle Broström, Jonatan D. Lindh, Buster Mannheimer

**Affiliations:** 1 Karolinska Institutet, Department of Clinical Science and Education at Södersjukhuset, Stockholm, Sweden; 2 Karolinska Institutet, Department of Laboratory Medicine, Division of Clinical Pharmacology, Karolinska University Hospital Huddinge, Stockholm, Sweden; University Hospital Llandough, UNITED KINGDOM

## Abstract

**Aims:**

Tramadol, a widely used analgesic drug, inhibits the reuptake of noradrenaline and serotonin impairing the aggregation function of thrombocytes. However, the risk for severe bleeding has previously not been studied. The aim of the present study is to investigate the association between tramadol and bleeding peptic ulcer in the Swedish population.

**Methods:**

In this register based case–control study based on the Swedish national patient registry and prescription drug registry, we included 18 306 patients hospitalized with a first-time diagnosis of bleeding peptic ulcer. For every case, 4 matched controls were included. To investigate the temporal aspects of tramadol induced bleeding ulcer, exposure was divided into patients with newly initiated and ongoing treatment. To explore a possible confounding by indication, the effect of codeine, a drug also prescribed for the treatment of moderate pain, but not known to affect thrombocyte function, was investigated. Univariable and multivariable logistic regression was used to analyse the association between tramadol use and bleeding ulcer.

**Results:**

Tramadol was associated with an increased risk of bleeding ulcer (adjusted odds ratio (aOR) 2.1, 95% confidence interval: (2.0–2.3). The association was stronger for newly initiated treatment with tramadol 2.8 (2.5–3.2) and weaker for ongoing treatment 1.7 (1.6–1.9). Codeine was also associated with an increased risk of bleeding ulcer 1.9 (1.7–2.1) and this association was also stronger for newly initiated treatment with codeine 2.3 (2.0–2.6) and weaker for ongoing treatment 1.7 (1.5–1.9).

**Conclusion:**

Treatment with tramadol was associated with an increased risk of bleeding peptic ulcer. Most of this association may be mediated by factors related to the pain condition rather than the pharmacologic effect per se.

## Introduction

About 10% of the Western world population will experience peptic ulcer at some point in their lives [1–4]. Bleeding peptic ulcer is associated with a substantial morbidity in terms of impaired quality of life, and cost for employers and health care systems [5]. The overall mortality rate is approximately 10% [6] and does not seem to decrease despite the introduction of endoscopic therapy [7]. After infection with Helicobacter pylori, drugs are the most common cause [8, 9], a problem that is likely to have increased during the last decades. According to a Swedish study, investigating drug dispensing from 2005 to 2008, the proportion of the population dispensed ≥5 drugs increased by 8.2%. The proportion of individuals exposed for excessive polypharmacy (≥10 drugs) increased even more [10]. Selective serotonin reuptake inhibitors (SSRIs) have been associated with bleeding peptic ulcer on the basis of thrombocyte inhibition [11]. Tramadol, a widely used analgesic drug also inhibits the reuptake of noradrenaline and serotonin, impairing the aggregation function of thrombocytes in a similar way, which in turn, may increase the risk for gastrointestinal bleeding [12]. However, evidence is lacking. The aim of the present study was to investigate the association between tramadol and bleeding ulcer in the Swedish population.

## Material and methods

### Study design and data sources

This case-control study used data from nationwide Swedish registries which were linked using the unique personal identification number assigned to all Swedish residents at birth or on a residence permit. Information from the National Patient Registry (NPR) [13, 14] was cross-linked with the Total Population Register (TPR) [15] and the Prescribed Drug Register (PDR) [16]. The NPR contains diagnostics codes on hospital inpatient care since 1987 and hospital outpatient care from 2001 onwards. The PDR contains information regarding drug dispensation in the entire Swedish population since 1 July 2005.

We classified the drugs by their ATC codes in the PDR register and diagnoses either as main or secondary diagnosis by their International Classification of Diseases (ICD-10 after 1996 and ICD-9 between 1987 and 1996) or ATC (Table 1). All diagnoses used for adjustment purposes were retrieved from both in- and outpatients from 1987 for both cases and controls. The diagnosis reflecting the outcome i.e. bleeding peptic ulcer were only searched among the inpatients during the study period.

**Table 1:** Definition of all variables included in the study.

Variables	Definition
**Outcome drug**	
Tramadol	ATC code beginning with: N02AX02
**Control drug**	
Codeine	ATC code beginning with: N02AA59, N02BE51, R05DA04, N02AA59, N02AJ06, N02AJ07, N02AJ08, N02AJ09, N02BE51 Drug name including “CITODON” or “PARACETAMOL/CODEINE”
**Drugs used to adjust the associations of the outcome and control drug**	One of the following ATC-codes beginning with:
Heparin	B01AB
NSAID	M01A, excluding M01AX05 and M01AH
Cox-2 inhibitors	M01AH
Acetylsalicylic acid (ASA)	B01AC06
Thrombocyte aggregation inhibiting agents	B01AC excluding B01AC06
Combinations including ASA	N02BA
Direct acting thrombocyte inhibiting agents	B01AE03, B01AE06, B01AE07, B01AF01, B01AF02
Other antithrombotic agents	B01AX
Warfarin	B01AA03
Corticosteroids	H02AB
Gastroprotective agents (GPA)	A02BA, A02BB, A02BC, A02BX
Selective serotonin reuptake inhibitors (SSRI)	N06AB, N06AX16, N06AX21, N06AA04
**Diseases used to adjust the associations of the outcome and control drug**	One of the following ICD-9 and ICD 10 codes beginning with:
Bleeding peptic ulcer	K250, K252, K254, K256, K260, K262, K264, KK266, K270, K272, K274, K276
Congestive heart failure	I500, I501, I509, 428A, 428B
Malignant disease	C000-C999, 140–209, 235–239
Chronic renal disease	N18, 585, 403
Liver disease / Cirrhosis	I982, I983, K701, K703, K704, K717, K721, K766, K767, 456B, 456C, 571C, 571D, 571F, 572D, 572E, 572W
	One of the following ATC- and ICD-codes, each beginning with:
Chronic obstructive pulmonary disease	ATC-code: R03BB04 ICD-9 and 10 codes: J44, 491C, 491W, 492, 496
Diabetes	ATC-code: A10 ICD-9 and 10 codes: E100 -E149, 250
Alcoholism	ATC-codes: N07BB01, N07BB03, N07BB04, N07BB05 ICD-9 and 10 codes: F10, 303, 291

The study was approved by the Regional Ethics Committee (Karolinska Institute, file record:13/3:7)

### Participants

#### Cases—With bleeding peptic ulcer

We defined the index date for the cases as the first time a patient occurred in the inpatient Swedish NPR between 1 July 2005 and 31 Dec 2012 due to a bleeding peptic ulcer either as main or secondary diagnosis. Individuals 18 years or older at the index date were eligible and patients with bleeding ulcer before 1 July 2005 were excluded. We then linked the cases to the NPR to receive diagnoses and to PDR to receive prescribed drugs.

#### Controls—Without bleeding peptic ulcer

For each case, we randomly selected 4 controls from the TPR register matched by gender and age within 2 years. Each control was only matched to one case and was not allowed to have had any previous documentation of bleeding ulcer. We then linked the controls to the NPR and PDR in the same way as for the cases.

### Exposures

We defined drug exposure as dispensing 90 days before the index date. The choice of 90 days was based on the Swedish regulation that most patient on long-term chronic treatment repeat their drug-dispensing every third month and on clinical experience. To investigate the temporal aspect of tramadol induced bleeding ulcer, exposure was divided into patients with newly initiated and ongoing treatment. Individuals with newly initiated drugs were those dispensed drugs within 90 days from index date but had not been dispensed such agents during the previous year (91–454 days before the index date). Individuals with repeated dispensations were the remaining group of patients that also received the drug 91–454 days from index date. To address a possible confounding by indication, the effect of codeine, a drug also prescribed for the treatment of moderate pain, but not known to affect thrombocyte function, was investigated in the same way as tramadol.

### Statistical methods

**Fig 1:**
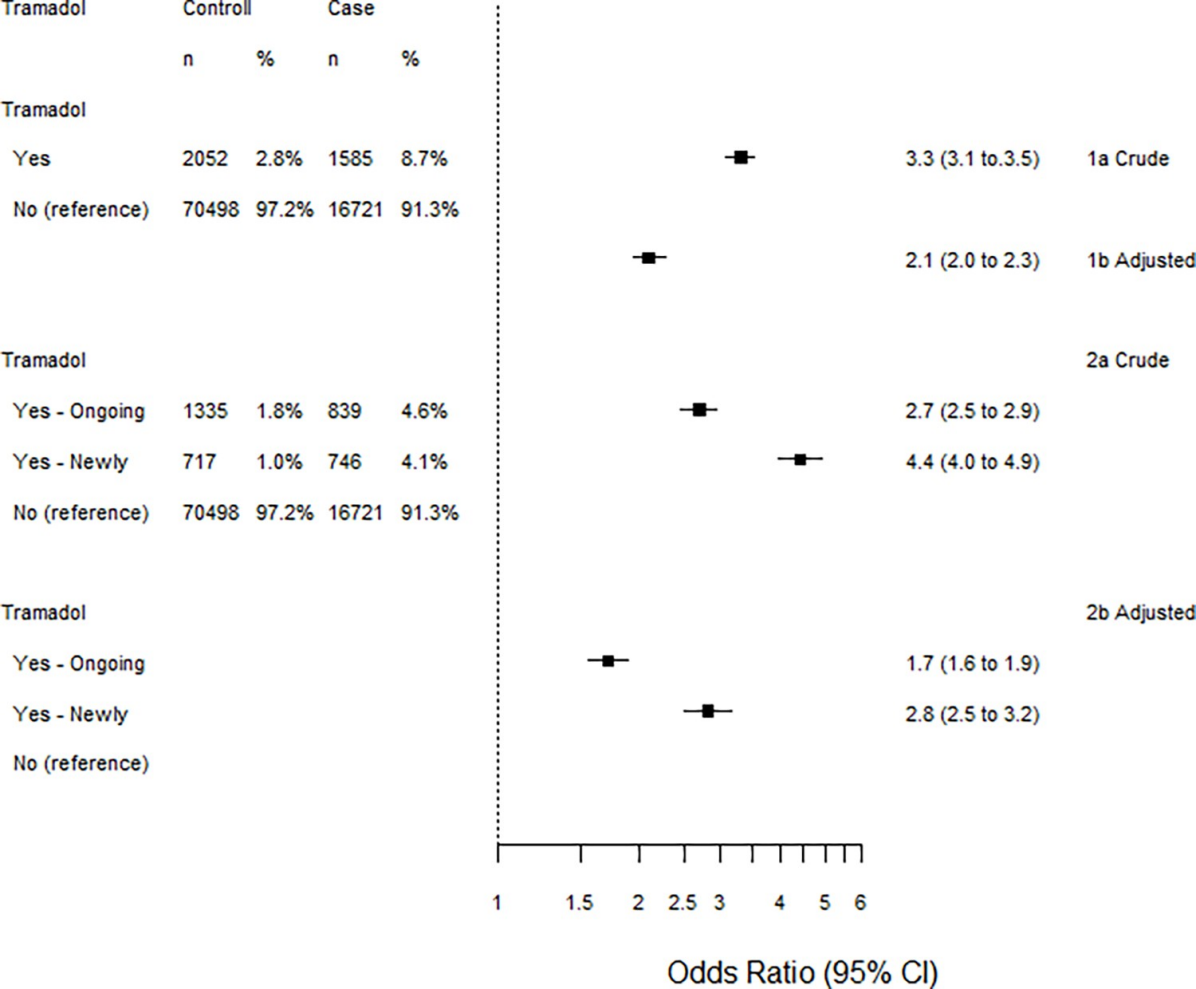
Associations between tramadol and bleeding peptic ulcers. Adjusted oddsratios (aOR) are adjusted for drugs and diagnoses in Table 2. The crude associations (1a and 2a) are higher compared with the adjusted associations (1b and 2b) for both definitions of prescription of tramadol (1a and 1b vs 2a and 2b). Newly prescription of tramadol (within 90 days and no treatment 91–454 days before) has the highest association with bleeding peptic ulcer (2a vs 1a and 2b vs 1b).

**Fig 2:**
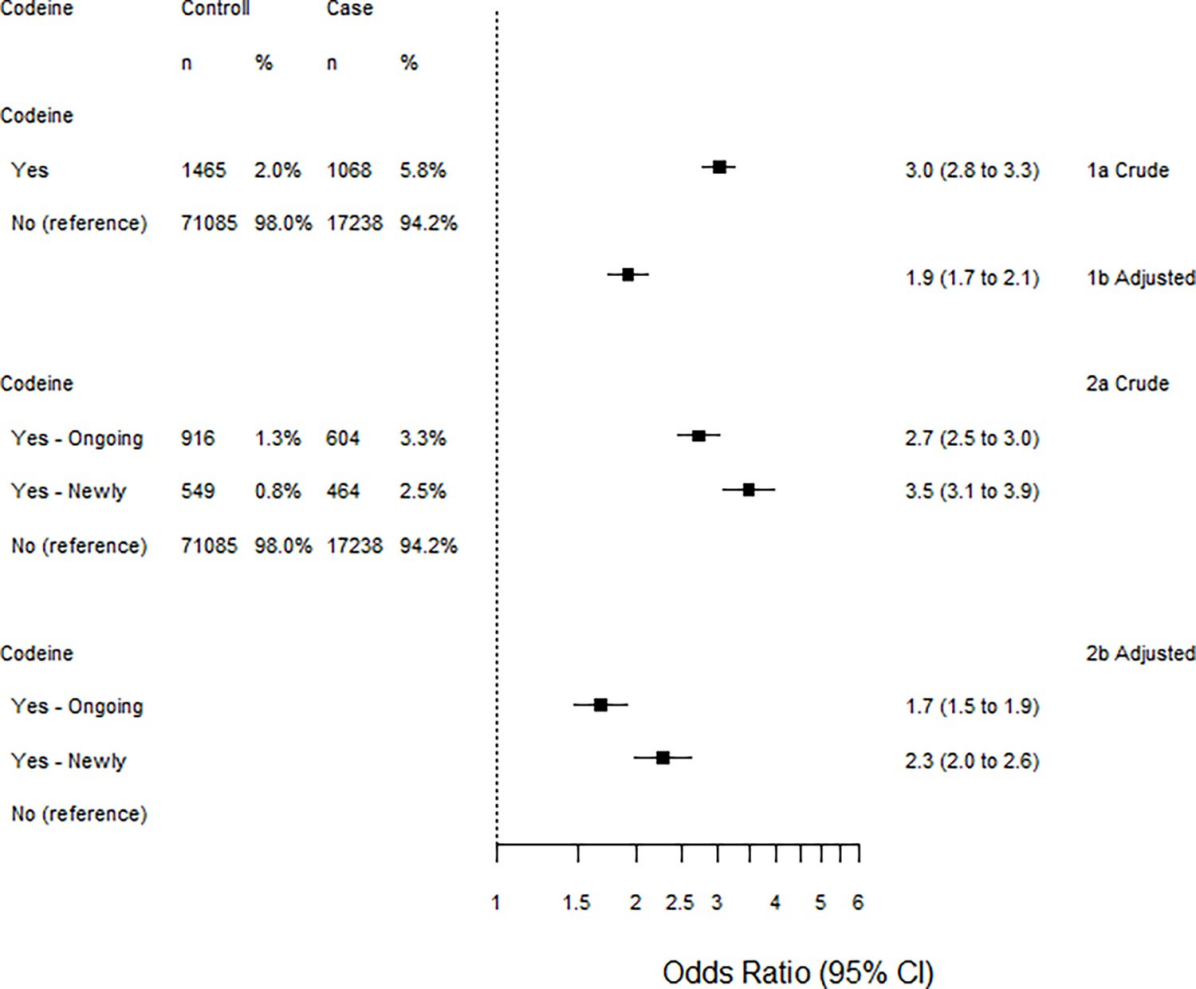
Associations between codeine and bleeding peptic ulcers. Adjusted oddsratios (aOR) are adjusted for drugs and diagnoses in Table 2. The crude associations (1a and 2a) are higher compared with the adjusted associations (1b and 2b) for both definitions of prescription of codeine (1a and 1b vs 2a and 2b). Newly prescriptions of codeine (within 90 days and no treatment 91–454 days before) has the highest association with bleeding peptic ulcer (2a vs 1a and 2b vs 1b).

We used conditional logistic regression to study the associations between tramadol and codeine and the risk of bleeding ulcer and to adjust the associations for differences regarding drugs and diseases between the cases and controls. Our model strategy was as follows: Firstly, we studied the unadjusted associations between bleeding ulcer and tramadol in univariable analysis (Fig 1: 1a Crude, 1b adjusted). To investigate the temporal effect of tramadol we investigated the associations after separating newly initiated drug treatment and ongoing treatment (2a Crude). Secondly, to adjust the association for drugs and diagnoses we added the variables in Table 1 in a multivariable analysis (2b adjusted). All univariable and multivariable analyses were adjusted for age, sex and time through the case-control matched design of the study. Results are presented as odds ratios (OR) and 95% confidence intervals (CIs) (Figs 1 and 2). All analyses were done in IBM SPSS version 23 and figures in R version 3.3.0, conditional logistic regressions were done using the cox regression procedure.

## Results

Over the study period, 18 306 individuals 18 years of age or older were hospitalized due to a first-ever entry of an ICD-10 code of bleeding peptic ulcer in Sweden. For every case, 4 matched controls were included (n = 72 550).

Mean (SD) age was 72 (15) years, 56.8% were males. Table 2 shows medical characteristics and treatments among the study population at index date. Overall, individuals with bleeding peptic ulcer had more diseases and dispensed more drugs compared with the controls without ulcer. The most commonly occurring drugs in both groups were acetylsalicylic acid, GPA and NSAID, while the most common diagnoses were malignant diseases and diabetes.

**Table 2:** Medical characteristics and type of treatment among cases and controls at index date included in the adjusted logistic regression analysis.

Drugs*	No bleeding peptic ulcer Controls (n = 72 550)		Bleeding peptic ulcer Cases (n = 18 306)	
	Number (%)		Number (%)	
Heparin	484	0.7%	622	3.4%
NSAID	4322	6.0%	3396	18.6%
COX-2 inhibitors	266	0.4%	226	1.2%
Acetylsalicylic acid (ASA)	15783	21.8%	6196	33.8%
Thrombocyte aggregation inhibiting agents	1860	2.6%	1041	5.7%
Combination including aspirin	215	0.3%	194	1.1%
Direct acting thrombocyte inhibiting agents	11	0.0%	21	0.1%
Other antithrombotic agents	1	0.0%	4	0.0%
Warfarin	3365	4.6%	1274	7.0%
Corticosteroids	2650	3.7%	1629	8.9%
Gastroprotective agents (GPA)	7529	10.4%	3066	16.7%
SSRI	5388	7.4%	2119	11.6%
Diagnoses**				
Congestive heart failure	5640	7.8%	2989	16.3%
Malignant disease	11570	15.9%	3804	20.8%
Chronic renal disease	885	1.2%	802	4.4%
Liver disease / Cirrhosis	81	0.1%	374	2.0%
Chronic obstructive pulmonary disease	3049	4.2%	1524	8.3%
Diabetes	8220	11.3%	3358	18.3%
Alcoholism	1424	2.0%	1499	8.2%

*Drugs dispensed 90 days before index date

**Diagnoses ever before index date.

Among the bleeding ulcer cases, 8.7% had been dispensed tramadol, of which 4.6 percentage units had an ongoing treatment and 4.1 percentage units were newly commencing treatment. Among the controls, only 2.8% had been dispensed tramadol. Of these 1.8 percentage units had ongoing and 1.0 percentage units had a newly initiated treatment (Fig 1). Tramadol was associated with bleeding ulcer (aOR 2.1, 95% CI: 2.0 to 2.3). For cases with newly dispensed tramadol treatment this association was stronger 2.8 (2.5 to 3.2) while it was weaker among cases with ongoing treatment 1.7 (1.6 to 1.9).

Codeine was also associated with bleeding ulcer (aOR 1.9, 95% CI: 1.7 to 2.1). The associations for newly initiated treatment 2.3 (2.0 to 2.6) and ongoing treatment 1.7 (1.5 to 1.9) with codeine were similar as for tramadol (Fig 2).

## Discussion

To our knowledge, this is the first study that investigates the associations between tramadol and bleeding peptic ulcer. Tramadol utilization was associated with a two-fold increased risk of bleeding ulcer in the Swedish adult population. The strength of this association among patients with newly initiated tramadol was higher compared with patients with ongoing treatment (aOR 2.8 vs 1.7).

The most important strength is the uniform health system in Sweden that enables the combining of nationwide complete registries to control for potential confounders. The study also has potential weaknesses. Most importantly, the observational case-control design makes it hard to control for confounding by indication. Although we did control for a large array of diseases and concomitant drugs there is always the risk of residual confounding. One such source is over the counter drugs, for example NSAIDs that are not registered in the prescribed drug register. Aiming to control for this, we used codeine as a comparator drug. Codeine is also used for the treatment of moderate pain but lacks the propensity to increase the risk of bleeding. Codeine is a prodrug and its pharmacological effect is dependent on O-demethylation to morphine [17]. Thus, there is no pharmacological basis for an increased risk for gastrointestinal bleeding. The association between codeine and bleeding ulcer seen in the present study suggest that the associations with bleeding ulcer may mostly be attributed to factors related to the pain condition rather than to respective substance per se. Although adjusting for most different drug groups and diagnoses that may associate with bleeding ulcer a certain degree of residual confounding cannot be excluded [18].

Tramadol, inhibits the reuptake of noradrenaline and serotonin impairing the aggregation function of thrombocytes which, in turn, may increase the risk for gastrointestinal bleeding [12]. Studies on the clinical effects of tramadol in this regard have up to now been lacking. However, SSRIs, are a group of drugs widely used for the treatment of depression and anxiety, and affect trombocytes in a similar fashion. The risk attributed by SSRIs for the development of gastrointestinal bleeding is well documented with an effect size comparable to the results of that of tramadol indicated in the present study. Thus, using a case control design, De Abajo et al. showed that newly initiated SSRIs increased the odds for gastrointestinal bleeding three-fold (OR 3). A Danish cohort study received similar results. Antidepressants that did not affect serotonin, were not associated with bleeding [19, 20].

The risk to be dispensed codeine was also almost two-fold (aOR 1.9) which indicates an increased risk associated with analgesics in general. In addition, for both tramadol and codeine, the risk among individuals with newly initiated drugs as compared to those with ongoing treatment was higher (OR 2.8 vs 1.7 and 2.3 vs 1.7 respectively). This discrepancy, with an increased risk for adverse effects seems plausible in the beginning of a new drug treatment and may support an effect attributed to the initiated substance per se [21]. An alternative explanation may be that the difference is due to imbalances between the two groups of patients regarding factors of importance for the development of gastrointestinal bleeding, i.e. confounding by indication. In essence, a novel need for analgesics, as opposed to a prolonged need, may be due to factors associated with bleeding.

Tramadol is a widely used drug, prescribed to approximately 2% of the Swedish population each year [22]. The described associations, regardless of underlying cause, may therefore have important clinical implications warranting vigilance concerning patients treated with tramadol.

In conclusion, treatment with tramadol was associated with an increased risk of bleeding peptic ulcer. Most of this association may be mediated by factors related to the pain condition rather than the pharmacologic effect per se.
